# Combination of standard axial and thin‐section coronal diffusion‐weighted imaging facilitates the diagnosis of brainstem infarction

**DOI:** 10.1002/brb3.666

**Published:** 2017-03-15

**Authors:** Philippe Felfeli, Holger Wenz, Mansour Al‐Zghloul, Christoph Groden, Alex Förster

**Affiliations:** ^1^ Department of NeuroradiologyUniversitätsmedizin MannheimUniversity of HeidelbergMannheimGermany

**Keywords:** axial, brainstem, coronal, diagnosis, diffusion‐weighted imaging, stroke

## Abstract

**Background and Purpose:**

Although diffusion‐weighted imaging (DWI) is a very sensitive technique for the detection of small ischemic lesions in the human brain, in particular in the brainstem it may fail to demonstrate acute ischemic infarction. In this study, we sought to evaluate the value of additional thin‐section coronal DWI for the detection of brainstem infarction.

**Methods:**

In 155 consecutive patients (median age 69 [interquartile range, IQR 57–78] years, 95 [61.3%] males) with isolated brainstem infarction, MRI findings were analyzed, with emphasis on ischemic lesions on standard axial (5 mm) and thin‐section coronal (3 mm) DWI.

**Results:**

On DWI, we identified ischemic lesions in the mesencephalon in 12 (7.7%), pons in 115 (74.2%), and medulla oblongata in 31 (20%) patients. In 3 (1.9%) cases—all of these with medulla oblongata infarction—the ischemic lesion was detected only on thin‐section coronal DWI. Overall, in 35 (22.6%) patients the ischemic lesion was more easily identified on thin‐section coronal DWI in comparison to standard axial DWI. In these, the ischemic lesions were significantly smaller (0.06 [IQR 0.05–0.11] cm^3^ vs. 0.25 [IQR 0.13–0.47] cm^3^; *p* < .001) in comparison to those patients whose ischemic lesion was more easily (6 [3.9%]) or at least similarly well identified (114 [73.5%]) on standard axial DWI.

**Conclusions:**

Since thin‐section coronal DWI may facilitate the diagnosis of brainstem infarction, we suggest its inclusion in standard stroke MRI protocols.

## 1 Introduction

Acute brainstem infarction accounts for approximately 10% of all acute ischemic strokes (de Ortiz, Alcala‐Galiano, Ochoa, Salvador, & Millan, 2013). Among these, pontine infarction is the most common type, followed by infarctions in the medulla oblongata, and the mesencephalon. The clinical presentation is highly variable including cranial nerve palsies, motor hemiparesis, sensory loss, ataxia, vertigo, or specific brainstem syndromes (de Ortiz et al., 2013). The main arterial supply of the brainstem arises from the vertebral arteries, anterior spinal artery, posterior inferior cerebellar arteries, basilar artery, anterior inferior cerebellar arteries, superior cerebellar arteries, and posterior cerebral arteries (Tatu, Moulin, Bogousslavsky, & Duvernoy, 1996). Most common causes of brainstem infarction comprise among others large vessel disease of the vertebral arteries or basilar artery, small vessel disease of small perforating arteries, and cardioembolism (de Ortiz et al., 2013).

Diffusion‐weighted imaging (DWI) is a very sensitive technique for detection of small ischemic lesions in the human brain and in particular in the posterior fossa (Wardlaw et al., 2000). Consequently, DWI has become a reliable mean to identify and secure the diagnosis of acute brainstem infarction (Toi et al., 2003). Nevertheless, even DWI may fail to demonstrate ischemic lesions in a substantial proportion of patients with brainstem infarction (Oppenheim et al., 2000; Sylaja, Coutts, Krol, Hill, & Demchuk, 2008).

In this study, we sought to evaluate the additional value of combined standard axial and additional thin‐section coronal DWI for the detection of brainstem infarction.

## 2 Patients and Methods

### 2.1 Patients

In this retrospective single‐center study, we identified all patients with isolated acute ischemic infarction in the brainstem from a MRI report database (2011–2015). The study was approved by the local institutional review board (Medizinische Ethikkommission II der Medizinischen Fakultät Mannheim).

### 2.2 MRI studies

Magnetic resonance imaging was performed on a 1.5‐T or a 3‐T MR system (Magnetom Sonata/Avanto/Trio, Siemens Medical Systems, Erlangen, Germany). A standardized protocol was used in all patients including standard axial and thin‐section coronal DWI. Parameters of DWI are displayed in Table 1.

**Table 1:** Sequence parameters of axial and coronal diffusion‐weighted imaging (DWI) at the department's MRI scanners

DWI Sequence	Parameters	MRI scanner		
		1.5‐T Siemens Sonata	1.5‐T Siemens Avanto	3‐T Siemens Trio
Axial	FOV	230 × 230	230 × 230	230 × 230
	Matrix	128 × 128	192 × 192	192 × 192
	ST	5	5	5
	Number of slices	24	24	24
	TR	4,400	4,000	4,000
	TE	101	96	91
	*b* Values	0, 1,000	0, 1,000	0, 1,000
Coronal	FOV	240 × 195	230 × 230	230 × 230
	Matrix	128 × 128	192 × 192	128 × 128
	ST	3	3	3
	Number of slices	20	20	20
	TR	3,300	3,400	3,600
	TE	101	96	107
	*b* Values	0, 1,000	0, 1,000	0, 1,000

FOV, field of view (mm × mm); ST, slice thickness (mm); TR, repetition time (ms); TE, echo time (ms), *b* values (s/mm^2^).

### 2.3 MRI analysis

Localizations of hyperintense lesions in the brainstem were noted on standard axial and thin‐section coronal DWI. The topography was determined according to the maps by Tatu et al. (1996) and categorized in (1) mesencephalon; (2) pons; and (3) medulla oblongata. The identifiability of ischemic lesions in standard axial and thin‐section coronal DWI was independently evaluated by two raters (P.F. and A.F. with 2 and 10 years experience in neuroimaging, respectively) and categorized as (1) better delineation on axial DWI; (2) better delineation on coronal DWI; (3) equal delineation on axial and coronal DWI. Cases with discrepancies were rereviewed by both readers and discussed until a consensus was reached. Ischemic lesion size was measured on DWI by manually delineated ROI, summation of these areas in cm^2^ on each section and multiplication with the slice thickness (plus interslice gap), to determine the volume in cm^3^ by use of OsiriX (Pixmeo SARL, Bernex, Switzerland; Rosset, Spadola, & Ratib, 2004).

### 2.4 Statistical analysis

All statistical analyses were performed using Statistical Product and Service Solutions (SPSS) statistics for Windows (Release 17.0; SPSS, Chicago, IL, USA). Normal distribution of the data was tested by use of the Kolmogorov–Smirnov test. Non‐normally distributed data are presented with median and interquartile range (IQR). Descriptive data were analyzed by use of chi‐square tests, or the Mann–Whitney *U* test as appropriate. Comparison of lesion size on DWI was performed using the Mann–Whitney *U* test. Interrater reliability regarding the identifiability of ischemic lesions on standard axial and thin‐section coronal DWI was assessed by Cohen's kappa coefficient. All statistics was performed with a .05 level of significance.

## 3 Results

Overall, we identified 155 consecutive patients (median age 69 [IQR 57–78] years, 95 [61.3%] males) with isolated brainstem infarction. On DWI, we identified ischemic lesions in the mesencephalon in 12 (7.7%), in the pons in 115 (74.2%), and in the medulla oblongata in 31 (20%) patients. Measurements of ischemic lesion size on thin‐section coronal DWI resulted in a smaller median volume compared to measurements on standard axial DWI in the mesencephalon (0.13 [IQR 0.06–0.16] cm^3^ vs. 0.14 [IQR 0.13–0.26] cm^3^, *p* = .09), pons (0.35 [IQR 0.18–0.56] cm^3^ vs. 0.47 [IQR 0.26–0.79] cm^3^, *p* < .001), and medulla oblongata(0.12 [IQR 0.06–0.15] cm^3^ vs. 0.14 [IQR 0.07–0.21] cm^3^, *p* = .02).

**Figure 1:**
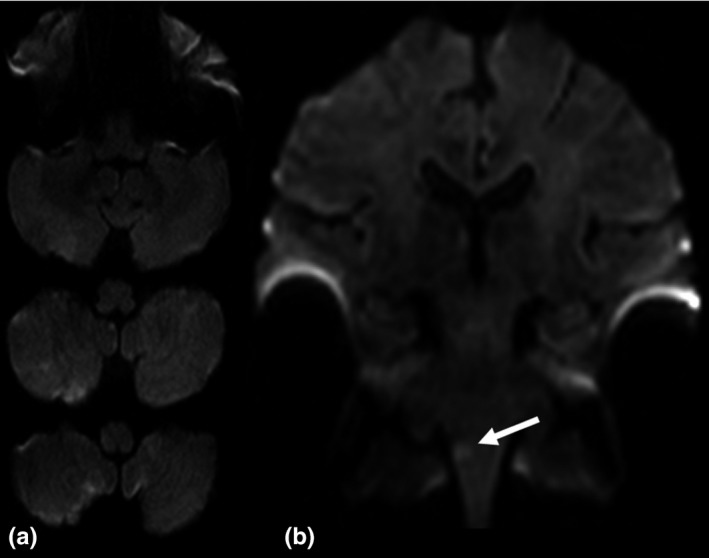
Acute ischemic infarction in the right medulla oblongata. (a) On standard axial diffusion‐weighted imaging (DWI) the ischemic lesion is not visible. (b) On thin‐section coronal DWI the ischemic lesion is clearly delineated (arrow)

**Figure 2:**
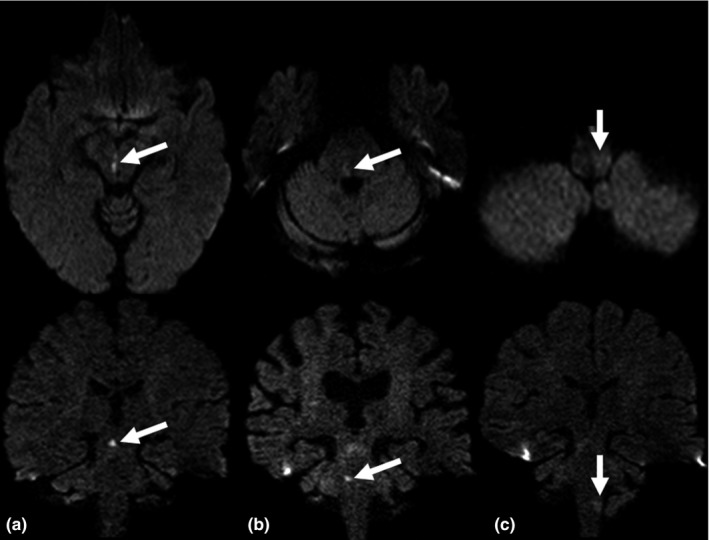
Examples of acute ischemic brainstem infarction more easily identifiable on thin‐section coronal diffusion‐weighted imaging (DWI) compared to standard axial DWI. (a) Mesencephalon. (b) Pons. (c) Medulla oblongata

In 3 (1.9%) cases—all these with medulla oblongata infarction—the ischemic lesion was detected only on thin‐section coronal DWI (for an example see Figure 1). Overall, in 35 (22.6%) patients the ischemic lesion was more easily identified on thin‐section coronal DWI in comparison to standard axial DWI. In detail, ischemic lesions were better identifiable on thin‐section coronal DWI in the mesencephalon in 3 (8.6%), in the pons in 16 (45.7%), and in the medulla oblongata in 16 (45.7%) patients. For example, see Figure 2. In these cases, the ischemic lesions were significantly smaller (0.06 [IQR 0.05–0.11] cm^3^ vs. 0.25 [IQR 0.13–0.47] cm^3^; *p* < .001) in comparison to those patients whose ischemic lesion was more easily (6 [3.9%]) or at least similarly well identified (114 [73.5%]) on standard axial DWI. The interrater reliability regarding the identification of ischemic lesions on standard axial and thin‐section coronal DWI was substantial (Cohen's kappa = .77, *p* < .001).

## 4 Discussion

Due to the brainstem's small size and its densely packed composition, a very small ischemic lesion may result in relevant clinical symptoms. Diffusion‐weighted imaging is the gold standard for the detection of acute ischemic stroke (Wardlaw et al., 2000). However, in patients with very small ischemic lesions especially in the posterior fossa even DWI may fail to demonstrate the pathology (Oppenheim et al., 2000; Sylaja et al., 2008). In order to overcome this limitation, additional thin‐section axial DWI of the infratentorium has been suggested (Entwisle, Perchyonok, & Fitt, 2016; Sorimachi, Ito, Morita, & Fujii, 2008). In this study, a thin‐section coronal DWI was added to the MRI protocol for patients with suspected brainstem infarction, since in coronal plane slices are positioned perpendicular to the course of the perforating brainstem arteries. Hereby, even very small ischemic lesions in the brainstem should be captured with greater certainty in comparison to DWI in axial or sagittal plane. In this study, in approximately 2% of cases the acute ischemic lesion has been demonstrated only by thin‐section coronal DWI. Although this number might appear low and perhaps even negligible on first glance, in the individual patient misdiagnosis may have serious consequences such as recurrent stroke (Kuruvilla, Bhattacharya, Rajamani, & Chaturvedi, 2011). Furthermore, very small ischemic lesions in the brainstem were much better identifiable on thin‐section coronal DWI in comparison to standard axial DWI. Thus, thin‐section coronal DWI might facilitate the detection of very small brainstem infarctions. With regard to the short acquisition time of DWI (approximately 1 min), inclusion of thin‐section coronal DWI in standard stroke MRI protocols should be considered in patients with suspected brainstem infarction to improve the detection of small and very small brainstem infarctions.

This study has some limitations. First, this is a retrospective clinical study of moderate size. However, to our knowledge this is the first series investigating the additional value of thin‐section coronal DWI for the diagnosis of acute brainstem infarction. Second, the study has been performed with different MRI scanners. However, DWI sequences have been customized for optimal comparability in daily clinical routine and consequently are generally comparable. Third, acute ischemic lesions on DWI were defined by a consensus reading and not by follow‐up MRI.

In conclusion, combination of standard axial and thin‐section coronal DWI possibly facilitates the diagnosis of brainstem infarction. Consequently, we suggest the inclusion of thin‐section coronal DWI in standard stroke MRI protocols for patients with suspected stroke in the posterior fossa.

## Conflict of Interest

None declared.
